# Identification of Metal Stresses in *Arabidopsis thaliana* Using Hyperspectral Reflectance Imaging

**DOI:** 10.3389/fpls.2021.624656

**Published:** 2021-02-16

**Authors:** Anne M. Ruffing, Stephen M. Anthony, Lucas M. Strickland, Ian Lubkin, Carter R. Dietz

**Affiliations:** ^1^Department of Molecular and Microbiology, Sandia National Laboratories, Albuquerque, NM, United States; ^2^Department of Computational Biology and Biophysics, Sandia National Laboratories, Albuquerque, NM, United States; ^3^Department of Electrical and Computer Engineering, Sandia National Laboratories, Albuquerque, NM, United States

**Keywords:** *Arabidopsis*, cesium stress, copper stress, hyperspectral imaging, metal stress, multivariate curve resolution, plant hyperspectral imaging, salt stress

## Abstract

Industrial accidents, such as the Fukushima and Chernobyl disasters, release harmful chemicals into the environment, covering large geographical areas. Natural flora may serve as biological sensors for detecting metal contamination, such as cesium. Spectral detection of plant stresses typically employs a few select wavelengths and often cannot distinguish between different stress phenotypes. In this study, we apply hyperspectral reflectance imaging in the visible and near-infrared along with multivariate curve resolution (MCR) analysis to identify unique spectral signatures of three stresses in *Arabidopsis thaliana*: salt, copper, and cesium. While all stress conditions result in common stress physiology, hyperspectral reflectance imaging and MCR analysis produced unique spectral signatures that enabled classification of each stress. As the level of potassium was previously shown to affect cesium stress in plants, the response of *A. thaliana* to cesium stress under variable levels of potassium was also investigated. Increased levels of potassium reduced the spectral response of *A. thaliana* to cesium and prevented changes to chloroplast cellular organization. While metal stress mechanisms may vary under different environmental conditions, this study demonstrates that hyperspectral reflectance imaging with MCR analysis can distinguish metal stress phenotypes, providing the potential to detect metal contamination across large geographical areas.

## Introduction

Anthropogenic activities or accidents associated with industrial processes may result in the release of toxic metals into the environment. Toxic metal exposure, particularly through groundwater contamination, poses a significant risk to human health. Additionally, metals in the environment may also impact plant, animal, and microbial life, resulting in damage to the ecosystem. Nuclear reactor accidents like the Chernobyl and Fukushima disasters released an estimated 149.8 petabecquerel (PBq) of radioactive cesium (Cs-134 and Cs-137) or approximately 47 kilograms (Imanaka et al., 2015). Due to the high water solubility of cesium, Cs-134 and Cs-137 spread rapidly in the environment and are taken up by local flora and fauna (Eikelmann et al., 1990; Ertel and Ziegler, 1991; Tsumune et al., 2013; Steinhauser et al., 2014). Conventional analytical techniques that require sample collection are ineffective at mapping Cs contamination from these events due to the large geographical areas of contamination. A rapid and non-invasive method for measuring environmental contamination by Cs and other toxic metals would reduce the risk of human exposure and facilitate cleanup efforts.

Metal stress in plants leads to changes in the natural photosynthetic pigments along with morphological changes in leaf structure. The pigment and morphological changes in vegetation may therefore serve as indirect indicators of metal contamination. Hyperspectral reflectance imaging has been applied to identify stress indicators in plants, including spectral signatures of metal contamination (Schuerger et al., 2003; Liu et al., 2010b; Li et al., 2015; Martinez et al., 2015; Shi et al., 2016; Zhang et al., 2017). These studies have generally focused on two different approaches for analyzing the reflectance spectra: (1) quantification of photosynthetic pigment concentration (chlorophyll-a, chlorophyll-b, carotenoids, or anthocyanins) from the spectral data (Blackburn, 2006), or (2) identification of specific spectral bands to provide indices for measuring metal stress (Horler et al., 1980; Liu et al., 2010b; Li et al., 2015; Martinez et al., 2015). While the first approach provides a direct link between the spectral data and plant physiology, it does not allow for a unique indicator of metal stress, as decreased chlorophyll or elevated carotenoid levels are general stress responses (Bertrand and Schoefs, 1999), and the pigment degradation products associated with specific stress responses may be unknown. Vegetation indices have successfully been applied for several decades in precision agriculture to assess crop health (Bannari et al., 1995), and new indices have been determined for metal stresses (Liu et al., 2010a,b; Martinez et al., 2015; Shi et al., 2016; Zhang et al., 2017). However, vegetation indices only utilize select wavelengths (typically 2–3) from the full spectra, increasing the odds of false positive identification, and in the case of metal stress indices, the wavelengths have not been correlated to a change in plant physiology.

While advancements in hyperspectral imaging technology now enable the collection of close-range hyperspectral datasets at high resolution, classification of disease or plant stress still often uses only a subset of those wavelengths. The advantage of hyperspectral imaging over multispectral imaging is that the subset of wavelengths to be employed need not be determined in advance. Instead, statistical approaches can be employed to determine which spectral bands offer the best classification (Lowe et al., 2017; Mishra et al., 2017). Instead of selecting a subset of features, feature extraction methods such as principal component analysis (PCA), independent component analysis (ICA), and multivariate curve resolution (MCR) analysis can transform the data into a new feature space with a reduced number of features (Mishra et al., 2017). Both methods of feature extraction, subsetting and transformation, reduce the effective dimensionality, where in the original, high-dimensional data significant amounts of redundant information were present (Mishra et al., 2017). Without such dimensionality reduction, the number of samples required to obtain statistical confidence for classification purposes would be prohibitive, growing exponentially with the dimensionality due to Hughes’ Phenomenon or the curse of dimensionality (Hughes, 1968; Thenkabail et al., 2014; Mishra et al., 2017). Since hyperspectral image data typically suffer from noise (Lowe et al., 2017), transformation feature extraction methods are often preferable over subset methods as use of the entire spectrum results in reduced noise. Among the feature extraction methods, MCR has the added advantage of returning spectra which are readily interpretable. Classification techniques, whether unsupervised techniques such as k-means clustering or supervised techniques including support vector machines, partial least squares-discriminant analysis, or artificial neural networks can then be employed on the reduced feature set (Mishra et al., 2017). This combination of close-range hyperspectral imaging and advanced analytics has been applied for drought stress and disease phenotyping (Renge and Mauring, 2013; Asaari et al., 2018; Bohnenkamp et al., 2019).

In this study, we applied hyperspectral reflectance imaging and MCR alternating least squares (MCR-ALS) analysis to identify unique spectral features associated with three stress phenotypes in the model plant *Arabidopsis thaliana*: salt, copper, and cesium stresses. To reduce variability in metal availability due to soil chelation, *A. thaliana* was grown hydroponically. Salt and copper stresses were included to determine whether cesium stress could be identified from other similar stresses. Common physiological measurements, such as root structure, leaf area, and cellular chloroplast organization, were acquired to assess the stress response. As cesium stress in *A. thaliana* has been shown to be influenced by the availability of potassium (Hampton et al., 2004), the effect of high and low potassium on the cesium stress response was also investigated. This study demonstrates the ability to distinguish between different types of stress responses based on hyperspectral reflectance imaging.

## Materials and Methods

### Materials

Seeds of *A. thaliana* Col-0 were obtained from the Lehle Seeds (WT-02). Chemicals were purchased from MP Biomedicals (KH_2_PO_4_ and ZnSO_4_^∗^7H_2_O), Sigma-Aldrich (CuCl_2_^∗^2H_2_O and CuSO_4_^∗^5H_2_O), Alfa Aesar (KNO_3_ and Agar, plant cell culture tested), and Fisher Scientific (all other chemicals).

### Growth and Metal Stress Treatment

*Arabidopsis thaliana* Col-0 were grown hydroponically, as described previously by Conn et al. (2013). Briefly, seeds were cold-acclimated at 4°C for 2 days on 0.7% germination medium agar plugs, partially submerged in 250 mL of germination medium. After cold acclimation, the gemination container was moved to a Shel Lab refrigerated diurnal plant growth chamber at 25°C under solar spectrum LED lights (Build My LED) at 60 to 105 μmol photons m^–2^s^–1^ light intensity with a 16:8 light:dark cycle. After roots had grown through the agar plug and into liquid medium, approximately 5–7 days, half (125 mL) of the germination medium was replaced with basal nutrient solution (Conn et al., 2013). After 24 h, all 250 mL of liquid growth medium was replaced with 250 mL of fresh basal nutrient solution. Plants then grew until they reached an appropriate size for hyperspectral imaging, approximately 5–7 more days. Plants with agar plugs were then moved to 50 mL centrifuge tubes with the conical end cut to allow for submersion in the liquid growth medium and a hole in the cap for holding the agar plug. Plants were randomly assigned to different experimental conditions and placed in a foam holder to keep the tubes afloat in 4.5 L of either normal or modified basal nutrient solution with air bubbling (Supplementary Figure 1). The modified basal nutrient solutions included an alternative concentration of one or more chemicals per condition. The conditions tested were: NaCl (25, 50, or 75 mM), CuCl_2_^∗^2H_2_O (25, 50, or 75 μM), CsCl (0.1, 1, or 10 mM), and CsCl (1 mM) with variable KCl (10 μM, 5.6 mM, or 25 mM) along with healthy controls for each experiment. Four to ten plants were grown in each condition per experiment, with most experiments containing five plants per condition.

### Confocal Fluorescence Microscopy

A leaf was cut from three different *A. thaliana* plants under each experimental condition. The leaves were placed on a glass microscope slide with the adaxial facing up. A No. 1.5 cover slip was pressed on top and taped flat. Each leaf was imaged using an Olympus IX71 confocal fluorescence microscope with a Disk Scanning Unit (DSU). The 60×/1.42 oil objective (∞/0.17/FN26.) was used to take all images. Images were acquired using a Q-imaging Rolera EM-C^2^ camera and Slidebook 6.0 software with exposure times of 1,000 ms, a gain of 2,500, intensification of 3,000, and using the chlorophyll fluorescence filter. For each leaf, 10 images were acquired across various regions of the leaf, each with 30 slices in the z-direction (step size = 0.1 μm). Microscopy was performed at days three and nine in the metal stress experiments. Data files were processed with ImageJ and merged.

### Quantification of Leaf and Root Areas

Leaf area for each *A. thaliana* plant was calculated from the hyperspectral reflectance images. During preprocessing of the hyperspectral reflectance data (described below), all plant pixels in each image were identified. Leaf area was then calculated by multiplying the total number of plant pixels by the measured pixel area (0.000112 cm^2^/pixel).

To quantify root area, each plant was moved to a 1 L polycarbonate Nalgene container containing approximately 800 mL of basal medium. A black sheet of paper was placed behind the container; a ruler was placed next to the container; and a digital image was acquired using a Cannon EOS Rebel T2i DSLR camera. In ImageJ, the scale was set for each image using the ruler in the image, and it was then cropped to only contain the white roots with the black background. The cropped images were converted to binary, and root area was calculated using the Analyze Particles function in ImageJ.

### Hyperspectral Reflectance Imaging

Hyperspectral images were obtained using a Hyperspec^®^ VNIR E-Series hyperspectral imager from Headwall Photonics equipped with a Cinegon 1.4/12 mm Compact C-Mount lens from Schneider-Kreuznach. A Spectralon puck with 99% reflectance (LabSphere) was used as the white reference for all images, and a black cap was placed over the lens for the dark reference. A white reference was taken at the beginning and end of each experiment, as well as in between conditions during each imaging session. Both the Spectralon target and the plants were placed horizontally onto a stand 1 ft. from the imager, illuminated by two equidistant, broad-spectrum halogen lights, which were allowed twenty minutes to warm up prior to each imaging session. Hyperspec III application software was used to capture images. The frequency of images (i.e., images every day or every other day) and duration of plant growth were dependent upon both condition and observable plant response.

### Preprocessing of Hyperspectral Reflectance Data

Preprocessing of hyperspectral reflectance data was performed by reading hyperspectral data files (reflectance data normalized to the white reference spectra) from the Headwall Photonics Hyperspec^®^ VNIR E-Series hyperspectral imager into a custom software application, executing an empirically derived algorithm on the reflectance data, and outputting a comma separated value file representing a plant mask of the hyperspectral reflectance data. In addition, the total count of identified plant pixels was also written to file.

The software algorithm formatted the reflectance data into a waveform spectrum for each pixel in the 2D hyperspectral reflectance image. For each pixel’s spectrum, the following properties were determined:

• Red Average: Average intensity of pixels between 750 and 850 nm.
• Orange Average: Average intensity of pixels between 550 and 600 nm.
• Blue Average: Average intensity of pixels between 410 and 450 nm.
• Blue-Green Average: Average intensity of pixels between 3 and 450 nm.
• Orange Average to Blue Average: Ratio of the Orange Average parameter to the Blue Average parameter.
• Red Average to Blue-Green Average: Ratio of the Red Average parameter to the Blue-Green Average parameter.
• Red Cutoff Threshold: Boolean flag indicating the average intensity of pixels between 750 and 850 nm is greater than 0.5 (referenced to the white reference spectra).

Once the properties for each pixel’s spectrum were calculated, the software algorithm determined a pixel to be that of a plant if the Red Cutoff Threshold was true, i.e., greater than 0.5, and the Orange Average to Blue Average was greater than 1.5 or the Red Average to Blue-Green Average was greater than 3. If a pixel was determined to be that of a plant, a “1” was written to a comma separated value file in the row, column corresponding to the X, Y position of the pixel. Otherwise, a “0” was written to the comma separated value file. In addition, if a pixel was determined to be that of a plant, the total number of plant pixels counter was incremented. The algorithm continued to analyze each pixel until all pixels in the hyperspectral reflectance image were evaluated. Once complete, the final count of the pixels counter was written to a file.

### Multivariate Curve Resolution Analysis

Sandia National Laboratories has developed software to efficiently analyze large hyperspectral images using MCR-ALS, which has previously been described (Van Benthem et al., 2002; Van Benthem and Keenan, 2004; Haaland et al., 2009; Jones et al., 2012). While the fundamental algorithms are as described in Jones et al. (2012), the graphical user interface (GUI) and workflow have been improved and are currently implemented in Matlab (version R2019b, MathWorks, Inc.). Critically, the current version is capable of analyzing arbitrarily large hyperspectral data matrices, limited only by the amount of memory installed on the computer; the datasets in this work were often tens of gigabytes. The MCR-ALS software also allows equality constraints to be applied to any variables or portions thereof (Van Benthem et al., 2002); this capability allows models to be built using portions of the data (e.g., control plants) and then later extended to additional conditions (e.g., metal stress).

MCR alternating least squares analysis was first applied to the control plants in order to determine the spectral components necessary to model normal growth of *A. thaliana*. The raw hyperspectral image data set, consisting of multiple dimensions (one spectral, two spatial, and additional dimensionalities corresponding to images of different plants at different times) were first unfolded into a two-dimensional matrix of individual spectra. Unfolding the data in this manner allowed information from all control plants throughout the entire experimental time course to be effectively combined for the determination of spectral components. After unfolding, the previously generated masks were applied to reduce the data matrix to only those image pixels within the masks (where the plant was present). To estimate the number of spectral components required, the eigenvalues of the hyperspectral data matrix were calculated using principal component analysis. The “elbow” in the scree plot of these eigenvalues then served as an estimate of the required number of spectral components. The estimated number of spectral components were initialized using randomly generated spectra and an additional baseline component (constant offset) was included, which allows offset correction. After performing MCR-ALS with the estimated number of components until convergence, the obtained spectral components and the principal components of the residuals were examined. If the estimated number of spectral components was too low, unmodeled signal is evident in the principal components of the residuals, whereas when the estimated number of components is too high, one or more of the spectral components appears as either a null component or pure noise. The number of spectral components employed was then adjusted, and MCR-ALS was rerun until a model was developed with an appropriate number of spectral components. As further discussed later, five spectral components were required to model the normal growth of *A. thaliana*, modeling both the plant and reflections from the background. For simplicity, these five components are collectively referred to as the control components.

Additional spectral components were then determined for each of the metal stress conditions, with the NaCl stress condition serving as an example. Another unfolded two-dimensional data matrix was generated containing the hyperspectral image data for the NaCl exposed plants across the entire experimental time course. The number of spectral components required to model this data was estimated as before. However, when the estimated number of spectral components were initialized along with the added baseline component, the first five spectral components were initialized with the control components while the remaining components were randomly initialized. Additionally, the control components were equality constrained so that their values remained constant throughout the MCR-ALS convergence process. As such, any additional components modeled aspects of the hyperspectral data which could not be modeled by the control components. MCR-ALS was run until it converged with an appropriate number of spectral components, where the number of spectral components employed was adjusted as previously described. In the case of NaCl stress, only one additional component was required. An identical process was performed for CuCl_2_, where again a single additional component was required, though the component was not identical to the one found for NaCl stress. When the same process was applied to the CsCl stress condition, two new spectral components were required. However, when MCR-ALS was applied to the hyperspectral data from plants where K^+^ was added in addition to CsCl, no additional spectral components were required beyond those employed for plants exposed to CsCl alone. For statistical analysis of the MCR analysis results, standard ANOVA analysis followed by Tukey-Kramer post-hoc testing would have been inappropriate due to observed heteroscedasticity (variance heterogeneity). Therefore, the Games-Howell test, an extension to the Tukey-Kramer post-hoc test to account for unequal variances, was used to identify which treatments have different mean concentration and the statistical significance (Games and Howell, 1976).

## Results

### Metal Stresses Lead to Changes in Biomass and Chlorophyll

For each metal stress in this study (NaCl, CuCl_2_, and CsCl), we varied the concentration of the metal stressor in the hydroponic growth medium to determine the concentration at which visible signs of stress (leaf necrosis or chlorosis) were visible. For NaCl, concentrations of 25, 50, and 75 mM were tested, and 75 mM of NaCl yielded consistent visible signs of stress in *A. thaliana*. For CuCl_2_, concentrations of 25, 50, and 75 μM were tested, and 75 μM of CuCl_2_ produced the most consistent stress response in *A. thaliana*. Lastly, CsCl concentrations of 0.1, 1, and 10 mM were tested, where concentrations of 1 mM or higher of CsCl resulted in reliable necrosis of hydroponically grown *A. thaliana*. Images from these concentration tests are shown in Supplementary Figure 2. This preliminary concentration screening identified the concentration of each stress treatment resulting in visible stress responses in *A. thaliana*.

**FIGURE 1:**
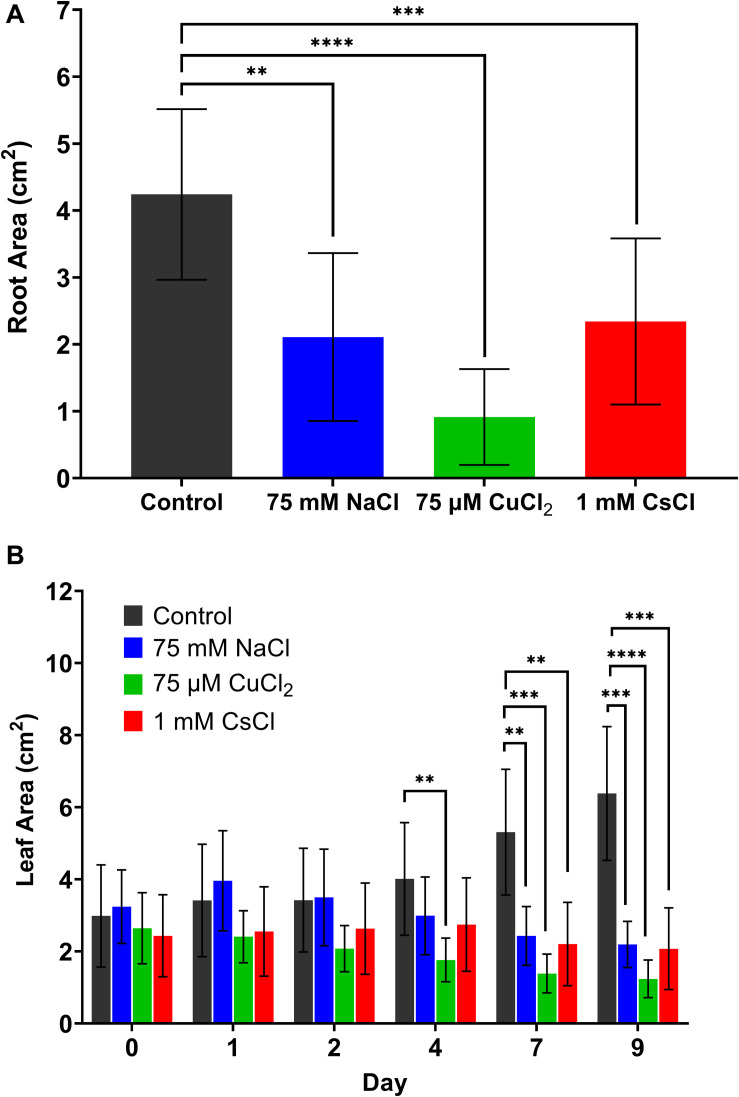
Changes in *A. thaliana* biomass with metal stress treatments. **(A)** root area after 9 days of metal stress treatment and **(B)** leaf area over time. All data are averages of at least five biological replicates with error bars indicating the standard deviation. Statistical significance determined by two-tailed *t*-test with equal variance comparing the treatment to the control; ^∗∗^*p* < 0.01, ^∗∗∗^*p* < 0.001, ^∗∗∗∗^*p* < 0.0001.

In order to compare different metal stress responses, the lowest concentration of each treatment that produced a visible stress response (75 mM of NaCl, 75 μM of CuCl_2_, and 1 mM of CsCl) was applied to hydroponically grown *A. thaliana* along with a control condition, with a total of five biological replicates under each condition. Both root and leaf areas were quantified to estimate changes in plant biomass resulting from each stress treatment. After 9 days of stress exposure, the root area of each plant was quantified using digital imaging and ImageJ pixel analysis, as described in subsection “Quantification of Leaf and Root Areas” of the methods section. As shown in Figure 1A, all metal stress conditions reduced root biomass compared to the control. The 75 μM CuCl_2_ condition showed the most significant reduction in root area, nearly 80% less than the control. Decreased root biomass was due to both a reduction in root length and lateral root structure (Supplementary Figure 3). Leaf area of each *A. thaliana* plant was quantified from the hyperspectral reflectance images of each plant, with image acquisition and image analysis described in subsections “Hyperspectral Reflectance Imaging” and “Quantification of Leaf and Root Areas” of the methods section (Figure 1B). While the healthy control plants showed increasing leaf area over the 9 days of growth, all metal stress treatments had either decreased or no change in leaf area. Reduced root and leaf biomass are common physiological indicators of stress in plants.

**FIGURE 2:**
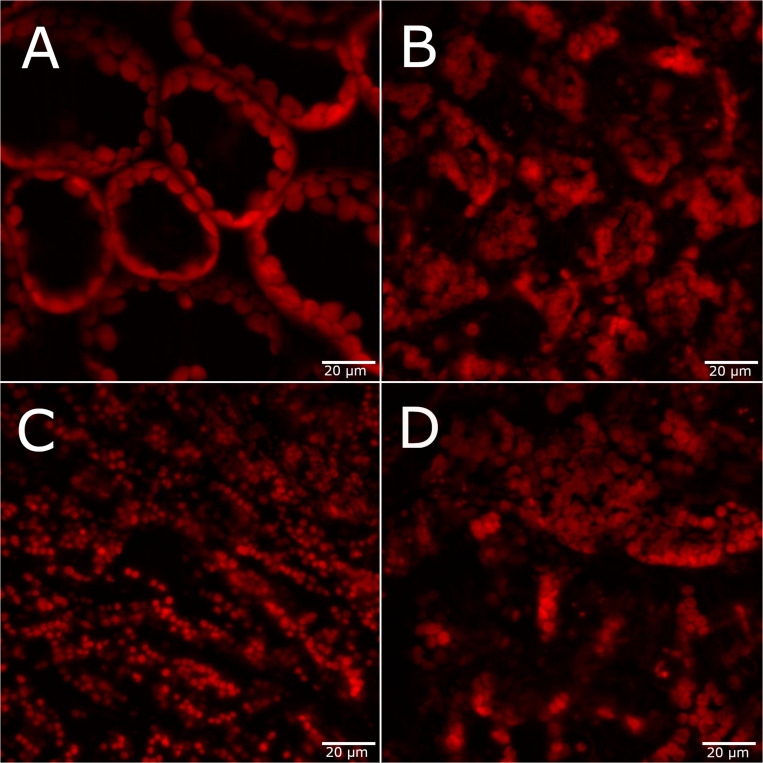
Confocal fluorescence microscopy images of *A. thaliana* leaves 4 days after stress exposure. **(A)** Control, **(B)** 75 mM NaCl, **(C)** 75 μM CuCl_2_, and **(D)** 1 mM CsCl conditions. Scale bar = 20 μm.

Changes in leaf chlorophyll content is another common physiological indicator of plant stress, and changes in photosynthetic pigments are the main contributors to spectral responses of plant stress. Therefore, confocal fluorescence microscopy images of chlorophyll fluorescence in the adaxial leaf surface were used to determine changes in chloroplast organization under each stress condition. Under the control condition, the chloroplasts surround the central vacuole with a spherical organization (Figure 2A). All metal stress conditions result in loss of the central vacuole and a disordered structure of the chloroplasts within the leaf tissue (Figures 2B–D). Under 75 mM NaCl conditions, the chloroplasts appear to be fuzzy, suggesting possible disruption of the chloroplast membrane due to osmotic stress. While there is considerable variability in the chlorophyll fluorescence images due to heterogeneity within and between each leaf, these features in chloroplast organization are observed in each treatment (see Supplementary Figure 4 for additional microscopy images of each condition).

### Metal Stresses Can Be Identified by Hyperspectral Reflectance Imaging

**FIGURE 3:**
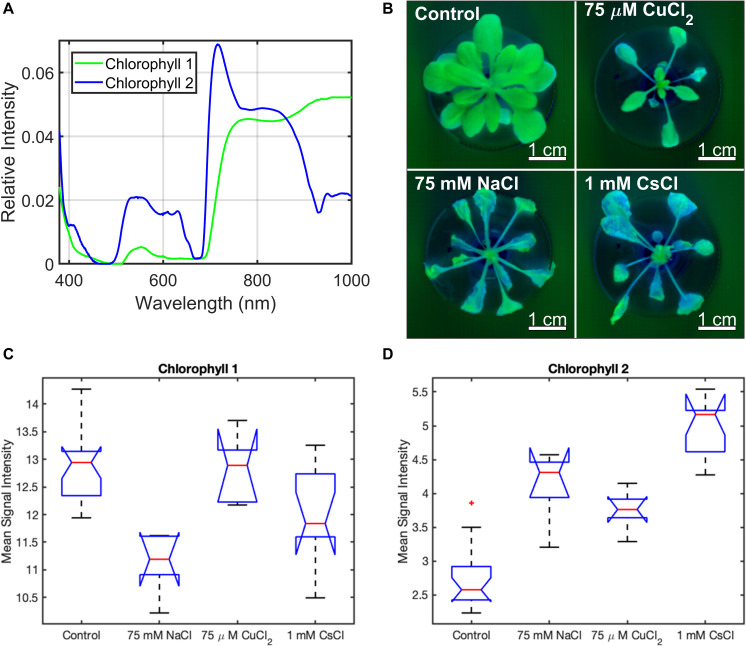
Chlorophyll spectral components of *A. thaliana* under different stress conditions from MCR analysis of hyperspectral reflectance data. **(A)** chlorophyll component spectra, **(B)** chl-1 (green) and chl-2 (blue) spectra in plant images under control and stress conditions, and **(C,D)** box and whisker plot of the mean signal intensity for the chl-1 **(C)** and chl-2 **(D)** components under control and stress conditions after 9 days of treatment. Mean signal intensity of the chlorophyll stress components is the average intensity of all plant pixels in an image for at least five biological replicates. For each box plot, the top and bottom of the box corresponds to the 25th and 75th percentile of the data, respectively, while the red line in the middle of the notch corresponds to the sample median across the replicates. The whiskers above and below each box show the extent of the data, aside from any outliers (marked with red asterisks). Observations are defined as outliers if they are more than 1.5 times the interquartile range away from the top or bottom of the box. Results of the Games-Howell test for statistical significance are shown in Table 1.

While *A. thaliana* showed common physiological stress responses to all metal treatments, the main goal of this study was to determine whether different stress responses can be identified solely from changes in their reflectance spectra. Hyperspectral reflectance images of each *A. thaliana* plant were collected across the 9 days of exposure to the control, 75 mM NaCl, 75 μM CuCl_2_, and 1 mM CsCl conditions, as described in subsections “Growth and Metal Stress Treatment” and “Hyperspectral Reflectance Imaging” of the methods section. The hyperspectral reflectance data were preprocessed and analyzed using MCR to identify the underlying component spectra as described in subsections “Preprocessing of Hyperspectral Reflectance Data” and “Multivariate Curve Resolution Analysis” of the methods section. Two chlorophyll components were identified under all conditions (chl-1 and chl-2) (Figure 3A). The healthy control and 75 μM CuCl_2_ treated plants contain higher levels of the chl-1 component, while all stress conditions have greater levels chl-2. Interestingly, the control plants show increased chl-2 component at the edges of the lower leaves (Figure 3B). The average signal intensities of chl-1 and chl-2 components for all plant pixels in the five biological replicates under each condition at day nine are shown in Figures 3C,D. This confirms that the observed changes in chlorophyll components from the images in Figure 3B are statistically significant. Signal intensity images and the mean signal intensities of chl-1 and chl-2 components over the entire timecourse of the experiment are included in Supplementary Figures 5, 6.

In addition to the chlorophyll components, MCR analysis masked to the plant pixels required three additional components to model the reflectance, even for the control condition. While necessary for modeling, these components are most likely due to reflectance (including multiple reflections) from the surroundings rather than from the plants as the average signal intensity for these components is higher in the background pixels than in the plant pixels. Additionally, a uniform offset baseline component was employed.

**FIGURE 4:**
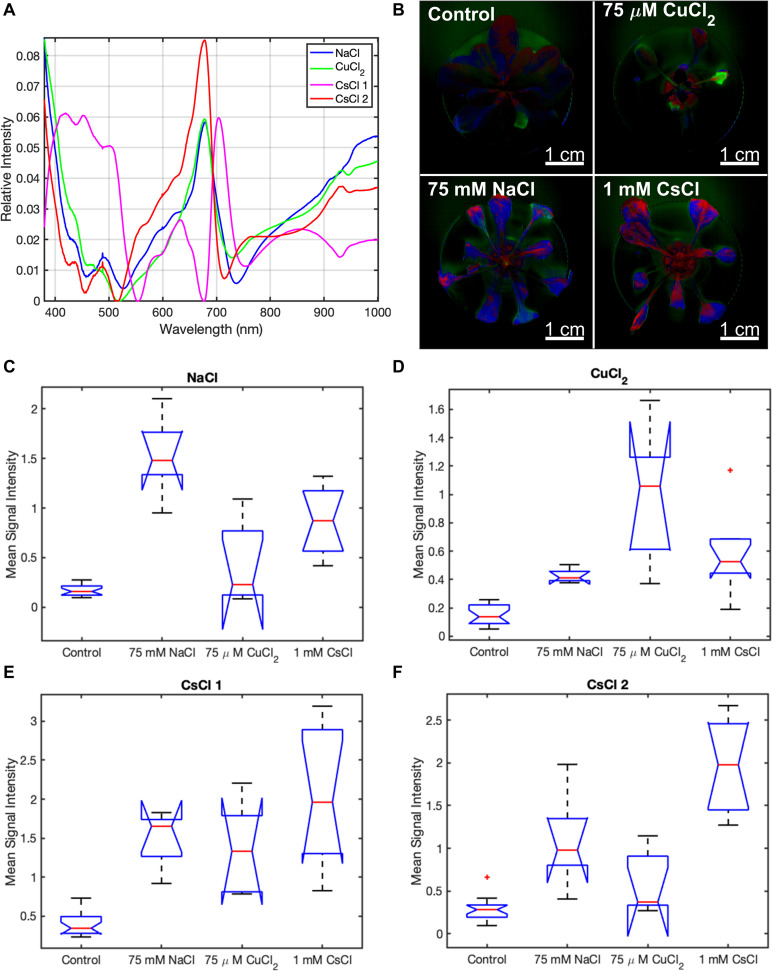
Stress spectral components of *A. thaliana* under different stress conditions from MCR analysis of hyperspectral reflectance data. **(A)** stress component spectra, **(B)** CuCl_2_ (green), NaCl (blue), and CsCl-2 (red) stress spectra in plant images under control and stress conditions, and **(C–F)** box and whisker plot of the mean signal intensity for the stress spectral components of NaCl **(C)**, CuCl_2_ **(D)**, CsCl-1 **(E)**, and CsCl-2 **(F)** under control and stress conditions after 9 days of treatment. Mean signal intensity of each stress component is the average intensity of all plant pixels in an image for at least five biological replicates. For each box plot, the top and bottom of the box corresponds to the 25th and 75th percentile of the data, respectively, while the red line in the middle of the notch corresponds to the sample median across the replicates. The whiskers above and below each box show the extent of the data, aside from any outliers (marked with red asterisks). Observations are defined as outliers if they are more than 1.5 times the interquartile range away from the top or bottom of the box. Results of the Games-Howell test for statistical significance are shown in Table 1.

While the combination of the baseline component, two chlorophyll components, and three background components appropriately modeled the control condition, MCR analysis required additional components to model each stress condition. For the NaCl and CuCl_2_ conditions, MCR analysis identified a single stress component for each condition, while analysis of the CsCl data yielded two components (Figure 4A). Of those two components, the first CsCl spectral component shows very little spectral similarity with the NaCl and CuCl_2_ spectral components (Figure 4A). The second CsCl spectral component is spectrally similar to the NaCl and CuCl_2_ spectral components, and its concentration was a more statistically significant indicator of CsCl stress. Therefore, only CsCl-2 component is included in the spectral images (Figure 4B). When the signal intensities of the stress spectra are mapped back onto the *A. thaliana* images, the control condition only has limited concentrations of the stress spectra primarily at the edges of the lower leaves (Figure 4B), indicating low levels of stress. For the stress conditions, all *A. thaliana* plants have pixels with each of the three stress components; however, the stress component associated with each condition is the predominant component in each image. The NaCl (blue) and CsCl-2 (red) stress components appear as major spectral components in both 75 mM NaCl and 1 mM CsCl images. However, the NaCl and CsCl-2 components show spatial differences. In the 75 mM NaCl condition, the NaCl component is located in the leaf tissue closer to the stem, while the CsCl-2 component is predominantly located in the leaf tips and severely discolored tissue. In the 1 mM CsCl condition, the CsCl-2 component is found in the leaf tissue near the stem and primary veins. Signal intensity images of the three stress components for all plants under the four experimental conditions are displayed in Supplementary Figure 7. The stress spectral component images provide qualitative evidence that CuCl_2_ stress can be distinguished from NaCl and CsCl stresses based on reflectance spectra.

For quantitative identification of stresses from hyperspectral reflectance data, Games-Howell statistical analysis was applied to the stress spectral signal intensities across all biological plant replicates (Table 1). Games-Howell is a post-hoc test for performing multiple comparisons. In many ways, the Games-Howell test provides results analogous to performing ANOVA followed by Tukey’s honestly significant difference (HSD) test. Both Games-Howell and Tukey’s HSD perform pairwise comparisons of the means (compare the mean of every condition to the mean of every other condition). While ANOVA followed by Tukey’s HSD is more commonly employed, our data clearly violates two of the necessary assumptions for this form of analysis, namely homogeneity of variances and equal sample sizes. The Games-Howell test is a straightforward extension of Tukey’s HSD to permit conditions to have differing variances and sample sizes, and hence it may be applied to our data.

**TABLE 1:** Games-Howell test results for statistical significance for all spectral components.

Spectral component	Conditions	*p*-value	Significance
**Chlorophyll-1**	Control vs. NaCl	0.0035	Significant
	Control vs. CuCl_2_	0.9993	Not Significant
	Control vs. CsCl	0.0572	Not Significant
	NaCl vs. CuCl_2_	0.0107	Significant
	NaCl vs. CsCl	0.1608	Not Significant
	CuCl_2_ vs. CsCl	0.2154	Not Significant
**Chlorophyll-2**	Control vs. NaCl	0.0097	Significant
	Control vs. CuCl_2_	0.0012	Significant
	Control vs. CsCl	0.0000	Significant
	NaCl vs. CuCl_2_	0.5581	Not Significant
	NaCl vs. CsCl	0.0888	Not Significant
	CuCl_2_ vs. CsCl	0.0004	Significant
**NaCl**	Control vs. NaCl	0.0062	Significant
	Control vs. CuCl_2_	0.5590	Not Significant
	Control vs. CsCl	0.0003	Significant
	NaCl vs. CuCl_2_	0.0148	Significant
	NaCl vs. CsCl	0.0706	Not Significant
	CuCl_2_ vs. CsCl	0.2783	Not Significant
**CuCl_2_**	Control vs. NaCl	0.0000	Significant
	Control vs. CuCl_2_	0.0607	Not Significant
	Control vs. CsCl	0.0025	Significant
	NaCl vs. CuCl_2_	0.1882	Not Significant
	NaCl vs. CsCl	0.3591	Not Significant
	CuCl_2_ vs. CsCl	0.3813	Not Significant
**CsCl-1**	Control vs. NaCl	0.0072	Significant
	Control vs. CuCl_2_	0.0737	Not Significant
	Control vs. CsCl	0.0007	Significant
	NaCl vs. CuCl_2_	0.9681	Not Significant
	NaCl vs. CsCl	0.3204	Not Significant
	CuCl_2_ vs. CsCl	0.3009	Not Significant
**CsCl-2**	Control vs. NaCl	0.1058	Not Significant
	Control vs. CuCl_2_	0.3687	Not Significant
	Control vs. CsCl	0.0000	Significant
	NaCl vs. CuCl_2_	0.4309	Not Significant
	NaCl vs. CsCl	0.0778	Not Significant
	CuCl_2_ vs. CsCl	0.0006	Significant
**NaCl**	Control vs. 1 mM CsCl, 25 mM KCl	0.1223	Not Significant
	Control vs. 1 mM CsCl, 5.6 mM KCl	0.0003	Significant
	Control vs. 1 mM CsCl, 10 μM KCl	0.0331	Significant
	1 mM CsCl, 25 mM KCl vs. 1 mM CsCl, 5.6 mM KCl	0.9991	Not Significant
	1 mM CsCl, 25 mM KCl vs. 1 mM CsCl, 10 μM KCl	0.9526	Not Significant
	1 mM CsCl, 5.6 mM KCl vs. 1 mM CsCl, 10 μM KCl	0.9759	Not Significant
**CuCl_2_**	Control vs. 1 mM CsCl, 25 mM KCl	0.4041	Not Significant
	Control vs. 1 mM CsCl, 5.6 mM KCl	0.0025	Significant
	Control vs. 1 mM CsCl, 10 μM KCl	0.0124	Significant
	1 mM CsCl, 25 mM KCl vs. 1 mM CsCl, 5.6 mM KCl	0.2387	Not Significant
	1 mM CsCl, 25 mM KCl vs. 1 mM CsCl, 10 μM KCl	0.0192	Significant
	1 mM CsCl, 5.6 mM KCl vs. 1 mM CsCl, 10 μM KCl	0.1103	Not Significant
**CsCl-1**	Control vs. 1 mM CsCl, 25 mM KCl	0.1780	Not Significant
	Control vs. 1 mM CsCl, 5.6 mM KCl	0.0007	Significant
	Control vs. 1 mM CsCl, 10 μM KCl	0.0407	Significant
	1 mM CsCl, 25 mM KCl vs. 1 mM CsCl, 5.6 mM KCl	0.1497	Not Significant
	1 mM CsCl, 25 mM KCl vs. 1 mM CsCl, 10 μM KCl	0.4089	Not Significant
	1 mM CsCl, 5.6 mM KCl vs. 1 mM CsCl, 10 μM KCl	0.9740	Not Significant
**CsCl-2**	Control vs. 1 mM CsCl, 25 mM KCl	0.1562	Not Significant
	Control vs. 1 mM CsCl, 5.6 mM KCl	0.0000	Significant
	Control vs. 1 mM CsCl, 10 μM KCl	0.0011	Significant
	1 mM CsCl, 25 mM KCl vs. 1 mM CsCl, 5.6 mM KCl	0.0007	Significant
	1 mM CsCl, 25 mM KCl vs. 1 mM CsCl, 10 μM KCl	0.0018	Significant
	1 mM CsCl, 5.6 mM KCl vs. 1 mM CsCl, 10 μM KCl	0.9999	Not Significant

The NaCl and CsCl-2 components were shown to be statistically significant for determining their respective conditions at the *p* < 0.05 level when examining the mean signal intensities across all plant pixels in the images (Figures 4C–F). In Figure 4D, the CuCl_2_ condition visually appears to have a much higher mean signal intensity of the CuCl_2_ component than the control condition, and in fact, the two conditions can be completely separated by a simple threshold at 0.3. However, due to the large variance for the CuCl_2_ condition, Games-Howell was not able to establish statistical significance for this particular comparison *p* = 0.06. When considering all pairwise comparisons between conditions, statistically significant (*p* < 0.05) differences between conditions could not always be established when examining only a single spectral component. However, highly statistically significant (*p* < 0.01) differences could be established between all pairs of conditions when employing simple combinations of the spectral components. Mean signal intensities for all stress spectra across the experimental timecourse are shown in Supplementary Figures 8–10.

### Increasing Potassium Concentration Reduces Cesium Stress

While these hydroponic metal stress experiments are conducted under controlled conditions, environmental samples will have significant variability in the chemical composition of the soil matrix. Previous studies have provided evidence that the mechanism of cesium toxicity in plants is due to either inhibition of potassium channels or functional effects on intracellular proteins that use potassium as a co-factor for folding or enzymatic activity (Hampton et al., 2004; Qi et al., 2008). To determine whether the spectral cesium stress response is dependent upon the level of potassium, we characterized the stress response of hydroponically grown *A. thaliana* to 1 mM of CsCl in the presence of low (10 μM), normal (5.6 mM), and high (25 mM) concentrations of potassium chloride (KCl), as described in subsection “Growth and Metal Stress Treatment” of the methods section.

**FIGURE 5:**
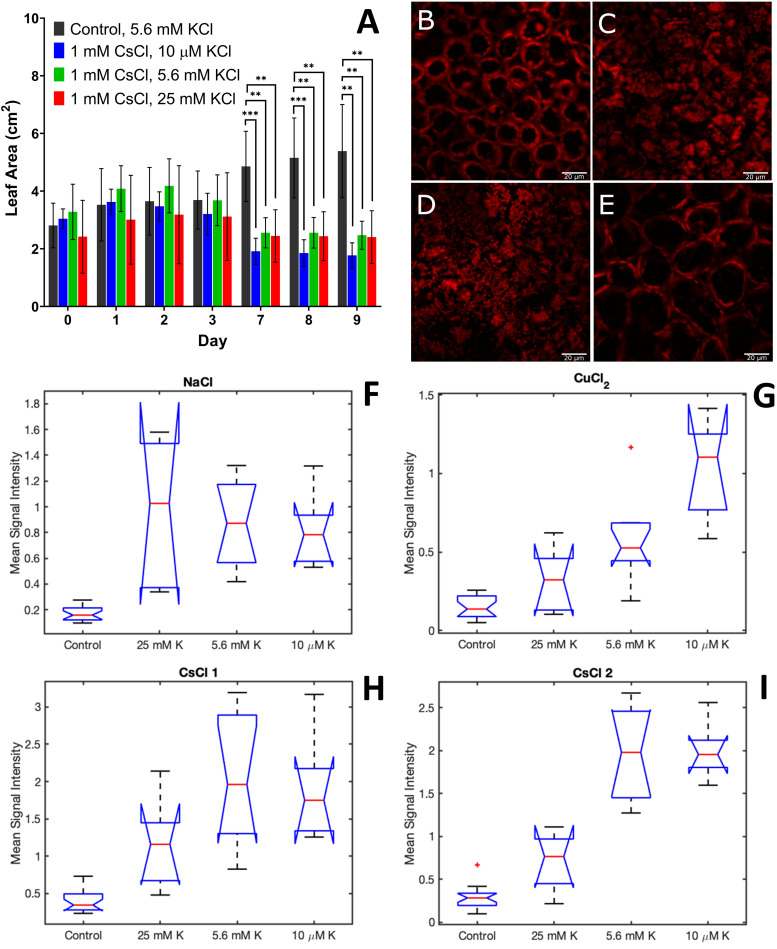
Physiological and spectral changes in *A. thaliana* exposed to 1 mM CsCl stress with varying levels of KCl. **(A)** leaf area; **(B–E)** confocal fluorescence microscopy images of *A. thaliana* leaves 9 days after exposure for control **(B)**, 1 mM CsCl with 5.6 mM KCl **(C)**, 1 mM CsCl with 10 μM KCl **(D)**, and 1 mM CsCl with 25 mM KCl **(E)**; and **(F–I)** mean signal intensity of NaCl **(F)**, CuCl_2_ **(G)**, CsCl-1 **(H)** and CsCl-2 **(I)** stress components 9 days after treatment. Leaf area is the average of at least five biological replicates with error bars indicating the standard deviation. Statistical significance for leaf area was determined by two-tailed *t*-test with equal variance comparing the treatment to the control; ^∗∗^*p* < 0.01, ^∗∗∗^*p* < 0.001. Scale bar for microscopy images is 20 μm. Mean signal intensities of the stress components are the average intensity of all plant pixels in an image for at least five biological replicates. For each box plot, the top and bottom of the box corresponds to the 25th and 75th percentile of the data, respectively, while the red line in the middle of the notch corresponds to the sample median across the replicates. The whiskers above and below each box show the extent of the data, aside from any outliers (marked with red asterisks). Observations are defined as outliers if they are more than 1.5 times the interquartile range away from the top or bottom of the box. Results of the Games-Howell test for statistical significance are shown in Table 1.

The physiological measurements of root area, leaf area, and chloroplast organization showed variable responses to 1 mM CsCl stress with changing potassium levels. Across all KCl levels, there was no significant change in root biomass (Supplementary Figure 11). However, leaf area was significantly reduced for all CsCl treatment conditions after 7 days of CsCl exposure, regardless of the level of potassium (Figure 5A). In contrast, confocal fluorescence microscopy showed that the addition of 25 mM KCl leads to retention of the central vacuole and chloroplast structural organization within the *A. thaliana* cells that is indistinguishable from the control without CsCl (Figures 5B,E and Supplementary Figure 12). Reducing potassium levels to 10 μM did not have a significant effect on chloroplast organization under Cs stress relative to the 5.6 mM KCl level (Figures 5C,D). These results suggest that some physiological changes of Cs stress, such as reduced leaf biomass, are independent of background K levels, while other Cs stress responses, such as chloroplast cellular organization, may be alleviated by increasing K.

The effect of variable K on the spectral response to CsCl stress was investigated using hyperspectral reflectance imaging and MCR analysis, as described in subsections “Hyperspectral Reflectance Imaging, Preprocessing of Hyperspectral Reflectance Data, and Multivariate Curve Resolution Analysis” of the methods section. The MCR results showed reduced levels of the CsCl-1 and CsCl-2 stress components with increased K levels of 25 mM (Figures 5H,I). For both CsCl spectral components, the pairwise comparisons of control-5.6 mM KCl and control-10 μM KCl were statistically significant (*p* < 0.01 for CsCl-2 and *p* < 0.05 for CsCl-1), while the pairwise comparison of control-25 mM KCl was not statistically significant (*p* > 0.05) (Table 1), providing additional evidence that increasing K levels reduces the spectral stress response to CsCl. Interestingly, low levels of K (10 μM KCl) resulted in increased CuCl_2_ spectra under 1 mM CsCl stress (Figure 5G), and while the NaCl stress component was elevated relative to the control, there was no significant change under variable KCl concentrations (Figure 5F).

## Discussion

The objective of this study was to determine whether spectral reflectance signatures of plants may provide unique indicators to distinguish between different types of metal stresses. The three stresses applied in this study – salt, copper, and cesium – have all been previously studied in *A. thaliana* (Zhu, 2000; Wójcik and Tukiendorf, 2003; Hampton et al., 2004; Le Lay et al., 2006; Wang et al., 2009; Zolla et al., 2009; Lequeux et al., 2010; Martínez-Peñalver et al., 2012; Burger et al., 2019). The significant decrease in root biomass (Figure 1A) and lateral roots (Supplementary Figure 3) under copper stress is in agreement with these previous studies (Wójcik and Tukiendorf, 2003; Lequeux et al., 2010). Reduced leaf biomass (Figure 1B) is also a commonly reported stress feature under all three stress conditions (Hampton et al., 2004; Lequeux et al., 2010; Ellouzi et al., 2011). While cesium stress was previously shown to lead to reduced chlorophyll-*a* and -*b* content in *A. thaliana*, particularly under conditions of depleted potassium (Le Lay et al., 2006), reduced chlorophyll content has also been detected under other stress conditions (Jansen et al., 2009; Martínez-Peñalver et al., 2012). These stress phenotypes were observed in the hydroponic metal stress treatments in this study, and while these serve as general stress indicators, they do not provide a unique signature for identification of the chemical stressor. This study demonstrated that MCR analysis of hyperspectral reflectance in the visible and near infrared (Vis-NIR) enables the identification of unique spectral components for the classification of stress phenotypes in *A. thaliana*. This capability represents an advancement over previous methods for stress identification using reflectance spectra which often rely on spectral indices of only a few wavelengths and have only demonstrated the ability to distinguish between healthy and a single stress condition (Liu et al., 2010b; Mahlein et al., 2013; Humplík et al., 2015; Li et al., 2015; Martinez et al., 2015). Using the full Vis-NIR reflectance spectra, this study distinguished between multiple stress conditions with similar phenotypes.

Multivariate curve resolution analysis of the hyperspectral reflectance data identified two chlorophyll spectral components (Figure 3A) and four stress-associated spectral components (Figure 4A). The chl-1 component shows common features for healthy plants with chlorophyll, including increased reflectance in the green region (520–560 nm) along with chlorophyll transparence near 700 nm, resulting in the “red edge” feature associated with cellular reflectance of infrared light (Figure 3A). In contrast, the chl-2 component contains a broader blue reflectance shoulder from 400 to 430 nm; greater green, yellow, and orange reflectance (500–640 nm); sharper chlorophyll absorbance features at 450–500 and 680 nm; a left shift and increased slope for the “red edge” between 690 and 705 nm; a dip following the red edge >710 nm; and reduced reflectance in the near infrared (910–940 nm). The left shift in the chlorophyll dip near 680 nm may indicate higher levels of chlorophyll-*b* in this component (Renge and Mauring, 2013), while the enhanced absorbance dip in the infrared indicates lower water content in the leaf tissue (Peñuelas et al., 1993). While the metal stress components share many common features, such as the peaks or shoulders at around 490 and 680 nm, there are clear differences across the three stress spectra. For example, the absorbance dips near 510 and 710 nm are shifted to the right for the NaCl spectrum; the CuCl_2_ spectral component has weaker absorbance dips at 450 and 715 nm; and the CsCl-2 spectral component has a stronger peak at 680 nm, a left shifted absorbance dip at 705 nm, and reduced reflectance in the near infrared (800–1000 nm). When the entire Vis-NIR reflectance spectrum is analyzed using MCR, these unique features within the stress spectral components can be used to distinguish between different types of metal stresses in *A. thaliana*.

Additional analytical techniques may be applied to improve the sensitivity of plant stress classification based on the hyperspectral reflectance response. For example, the localization of stress component spectra within the plant may be an important factor. In Figure 4B, the CsCl-2 stress spectrum has higher abundance near the primary leaf vein under the 1 mM CsCl condition yet is primarily in the leaf tips under the 75 mM NaCl condition. Similar localization is observed for the NaCl stress component spectra, which shows higher concentrations near the primary leaf vein under the 75 mM NaCl condition. Therefore, higher weighting of the stress component spectra near the primary leaf vein may improve the classification.

While MCR-ALS analysis of Vis-NIR reflectance enabled stress classification in *A. thaliana*, additional testing and improvements are likely necessary before this technology may be applied to identify metal contamination in the environment. Multiple plant species must be tested beyond *A. thaliana* to determine whether the stress component spectra are conserved across plant species. The conservation of spectral response will be linked to the underlying stress mechanisms. Therefore, plant species with different tolerances to the stress condition will have variable concentration-dependent responses, yet conserved mechanisms, such as the inhibition of potassium transport or function by cesium, may produce similar spectral responses. Furthermore, environmental vegetation may be subject to multiple stresses simultaneously; the effect of multiple stresses on the plant spectral response must also be investigated. While spectral-based identification of plant stresses may be applied empirically, understanding the physiological mechanism associated with the unique spectral response will allow for a comprehensive understanding of plant stress responses and possibly mechanisms for stress tolerance.

This study demonstrates that hyperspectral reflectance in the Vis-NIR can be used to identify stresses in *A. thaliana* through the application of MCR analysis. This technology may be applied to track the spread of chemical contaminants in the environment, such as the release of radioactive Cs from nuclear power plant accidents, and it may also be used in precision agriculture to detect nutrient limitations or crop diseases that produce a spectral response (Lee et al., 2010).

## Data Availability Statement

The raw data supporting the conclusions of this article will be made available by the authors, without undue reservation.

## Author Contributions

AR conceived and designed this study and established all experimental protocols. SA analysed the hyperspectral reflectance data. LS and IL conducted the growth experiments, collected the hyperspectral data, performed the microscopy and analysis, and analysed the root area. CD developed the method for quantifying leaf area and performed preprocessing of the hyperspectral data. AR and SA wrote the introduction, results, and discussion sections. All authors contributed to writing the methods section, reviewed, and approved the manuscript.

## Disclaimer

This paper describes objective technical results and analysis. Any subjective views or opinions that might be expressed in the paper do not necessarily represent the views of the U.S. Department of Energy or the United States Government.

## Conflict of Interest

The authors declare that the research was conducted in the absence of any commercial or financial relationships that could be construed as a potential conflict of interest.
