# Supplementary material for: Identification of Metal Stresses in Arabidopsis thaliana Using Hyperspectral Reflectance Imaging
Source: Front Plant Sci. 2021 Feb 16;12:624656. doi: 10.3389/fpls.2021.624656 (PMC7921809; doi:10.3389/fpls.2021.624656)
Supplement: Supplementary file 1 [file Data_Sheet_1.DOCX]

Supplementary Material

**Identification of Metal Stresses in *Arabidopsis thaliana* Using Hyperspectral Reflectance Imaging**

# Supplementary Figures (S1 and S2)

**Figure S1.** Hydroponic growth of *A. thaliana*. (A) germination growth chamber and (B) metal stress treatment growth chamber.


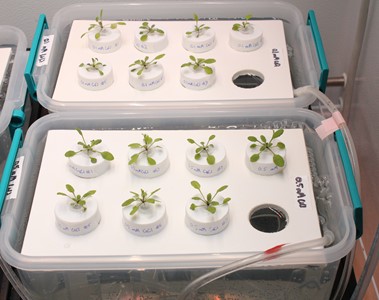

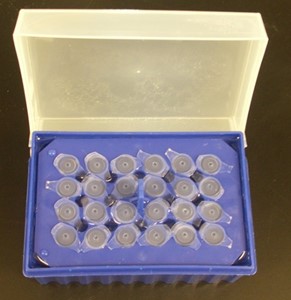


**A**

**B**


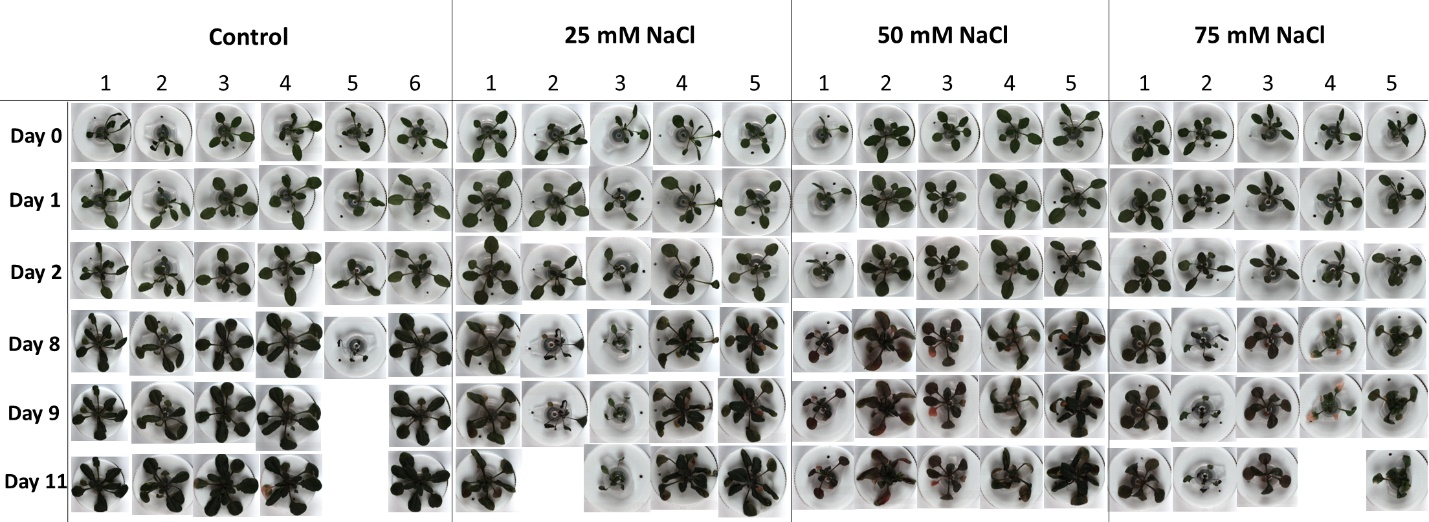


**B**

**A**


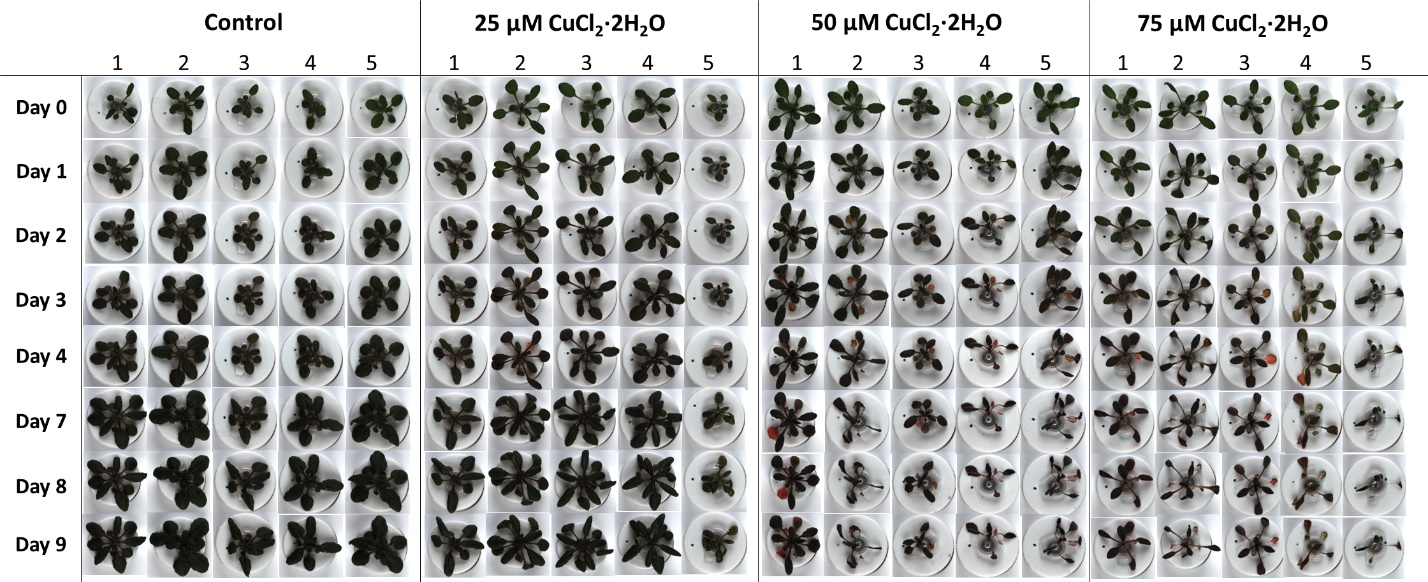


**C**


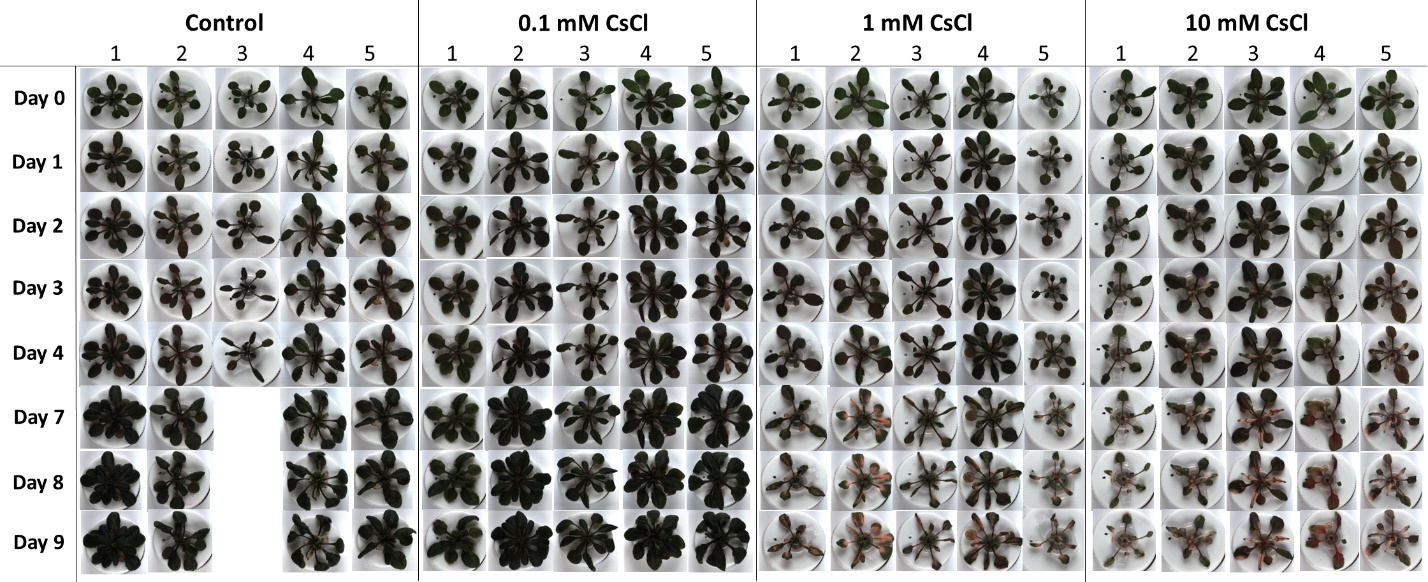


**Figure S2.** Color (RGB) images of *A. thaliana* under metal stress treatments: (A) NaCl, (B) CuCl_2_, and (C) CsCl.

# Supplementary Figure Captions (S3 – S12)

See separate files for images that were too large to include in this supplementary file. Captions are provided below.

**Figure S3.** Root images of *A. thaliana* grown hydroponically with control and metal stress treatments five days after stress exposure (scale bar = 1 cm).

**Figure S4.** Confocal fluorescence microscopy images of *A. thaliana* leaves under control and metal stress treatments, four days after exposure. Scale bar = 20 µm.

**Figure S5.** Signal intensity images of chl-1 (green) and chl-2 (blue) stress spectra under each environmental condition: control (A), 75 mM NaCl (B), 75 µM CuCl_2_ (C), and 1 mM CsCl (D).

**Figure S6.** Mean signal intensities of chl-1 (A, B, C, D) and chl-2 (E, F, G, H) spectral component for at least 5 biological replicates for control (A,E), 75 mM NaCl (B,F), 75 µM CuCl_2_ (C,G), and 1 mM CsCl (D,H) conditions. For each box plot, the top and bottom of the box corresponds to the 25^th^ and 75^th^ percentile of the data respectively while the red line in the middle of the notch corresponds to the sample median across the replicates. Notches which do not overlap have statistically significant differences in the median at the p < 0.05 significance level. The whiskers above and below each box show the extent of the data, aside from any outliers (marked with red asterisks). Observations are defined as outliers if they are more than 1.5 times the interquartile range away from the top or bottom of the box.

**Figure S7.** Signal intensity images of the NaCl, CuCl_2_, and CsCl-2 stress spectra under each environmental condition: control (A), 75 mM NaCl (B), 75 µM CuCl_2_ (C), and 1 mM CsCl (D).

**Figure S8.** Mean signal intensities of NaCl spectral component for at least 5 biological replicates for control (A), 75 mM NaCl (B), 75 µM CuCl_2_ (C), and 1 mM CsCl (D) conditions. For each box plot, the top and bottom of the box corresponds to the 25^th^ and 75^th^ percentile of the data respectively while the red line in the middle of the notch corresponds to the sample median across the replicates. Notches which do not overlap have statistically significant differences in the median at the p < 0.05 significance level. The whiskers above and below each box show the extent of the data, aside from any outliers (marked with red asterisks). Observations are defined as outliers if they are more than 1.5 times the interquartile range away from the top or bottom of the box.

**Figure S9.** Mean signal intensities of CuCl_2_ spectral component for at least 5 biological replicates for control (A), 75 mM NaCl (B), 75 µM CuCl_2_ (C), and 1 mM CsCl (D) conditions. For each box plot, the top and bottom of the box corresponds to the 25^th^ and 75^th^ percentile of the data respectively while the red line in the middle of the notch corresponds to the sample median across the replicates. Notches which do not overlap have statistically significant differences in the median at the p < 0.05 significance level. The whiskers above and below each box show the extent of the data, aside from any outliers (marked with red asterisks). Observations are defined as outliers if they are more than 1.5 times the interquartile range away from the top or bottom of the box.

**Figure S10.** Mean signal intensities of CsCl-2 spectral component for at least 5 biological replicates for control (A), 75 mM NaCl (B), 75 µM CuCl_2_ (C), and 1 mM CsCl (D) conditions. For each box plot, the top and bottom of the box corresponds to the 25^th^ and 75^th^ percentile of the data respectively while the red line in the middle of the notch corresponds to the sample median across the replicates. Notches which do not overlap have statistically significant differences in the median at the p < 0.05 significance level. The whiskers above and below each box show the extent of the data, aside from any outliers (marked with red asterisks). Observations are defined as outliers if they are more than 1.5 times the interquartile range away from the top or bottom of the box.

**Figure S11.** Root images of *A. thaliana* grown hydroponically with control and 1 mM CsCl stress treatments with varying levels of KCl, nine days after stress exposure (scale bar = 1 cm).

**Figure S12.** Confocal fluorescence microscopy images of *A. thaliana* leaves under control and 1 mM CsCl stress treatments with varying levels of KCl, 9 days after exposure. Scale bar = 20 µm.
