# Supplementary figures and images for: Identification of Metal Stresses in Arabidopsis thaliana Using Hyperspectral Reflectance Imaging
Source: Front Plant Sci. 2021 Feb 16;12:624656. doi: 10.3389/fpls.2021.624656 (PMC7921809; doi:10.3389/fpls.2021.624656)

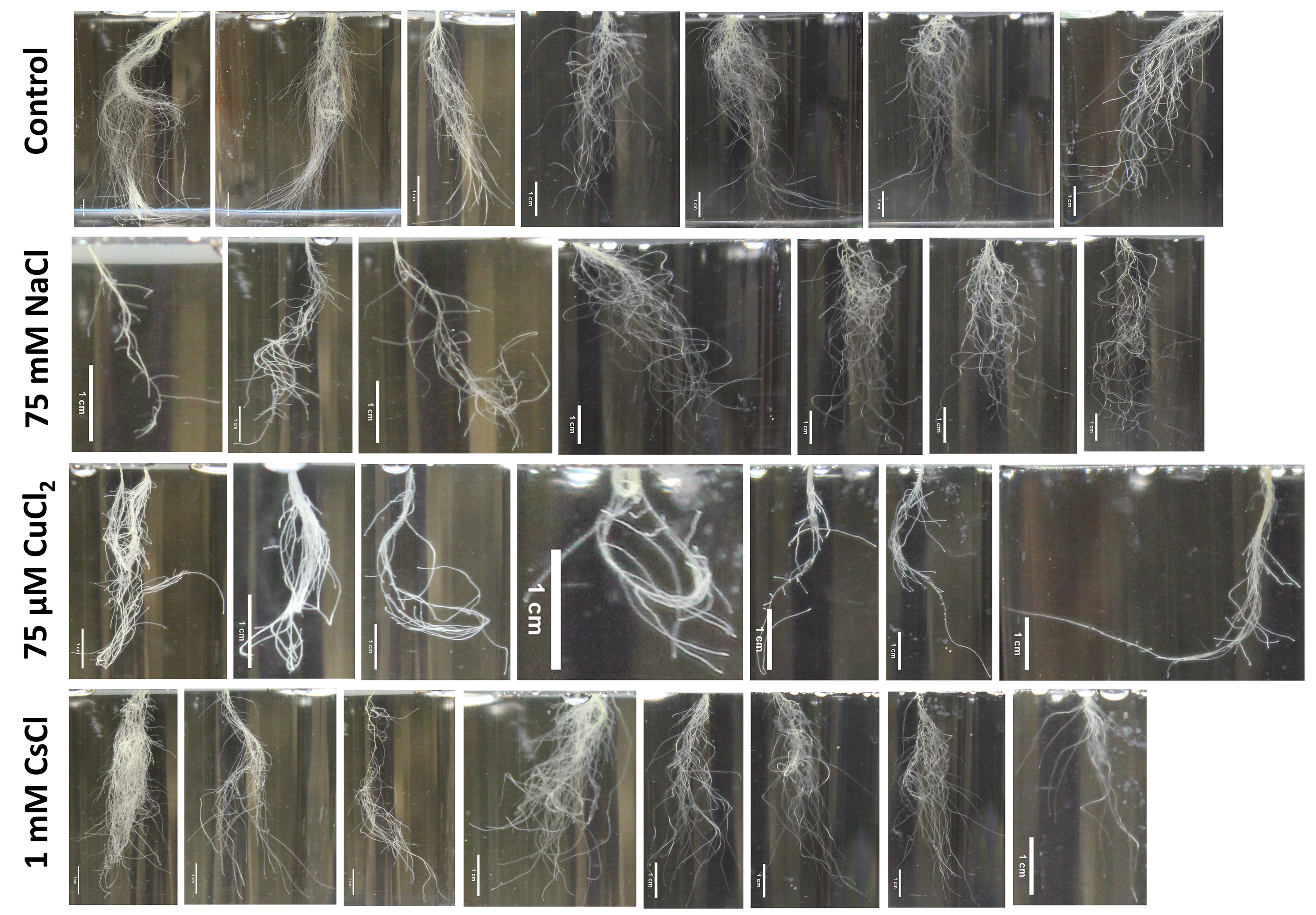

Supplement: Supplementary file 2 [file Image_3.tif]

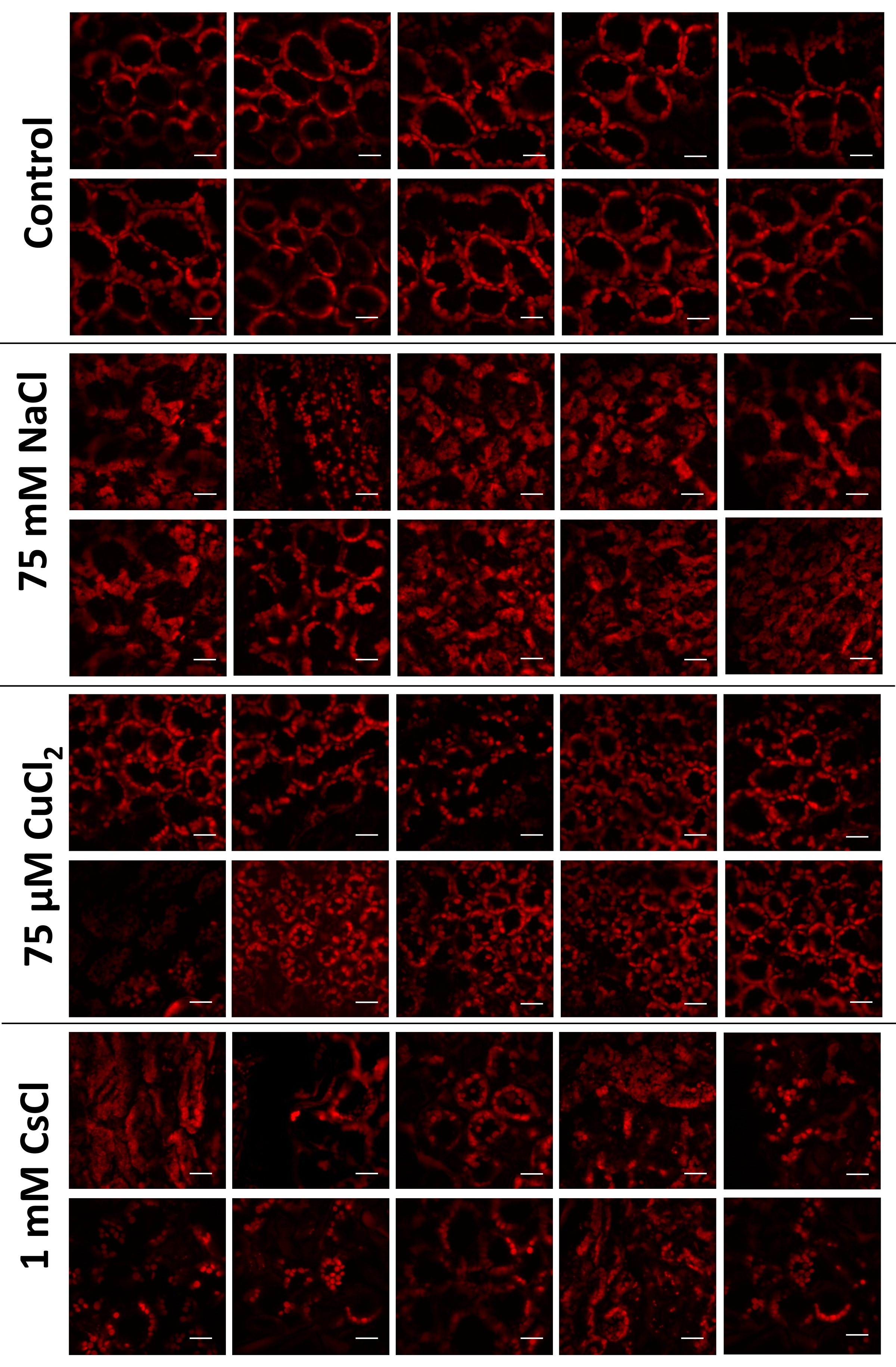

Supplement: Supplementary file 3 [file Image_4.tif]

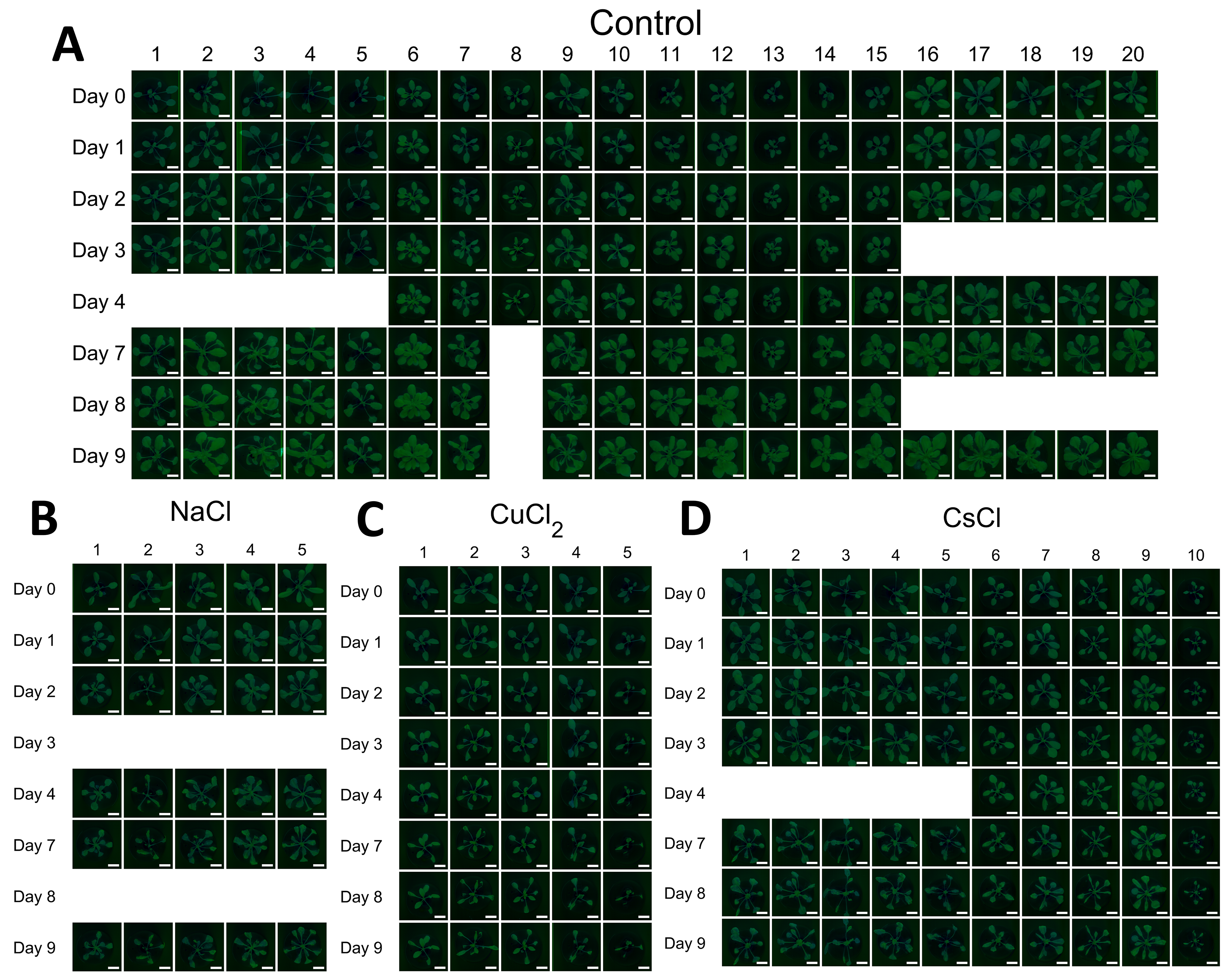

Supplement: Supplementary file 4 [file Image_5.tif]

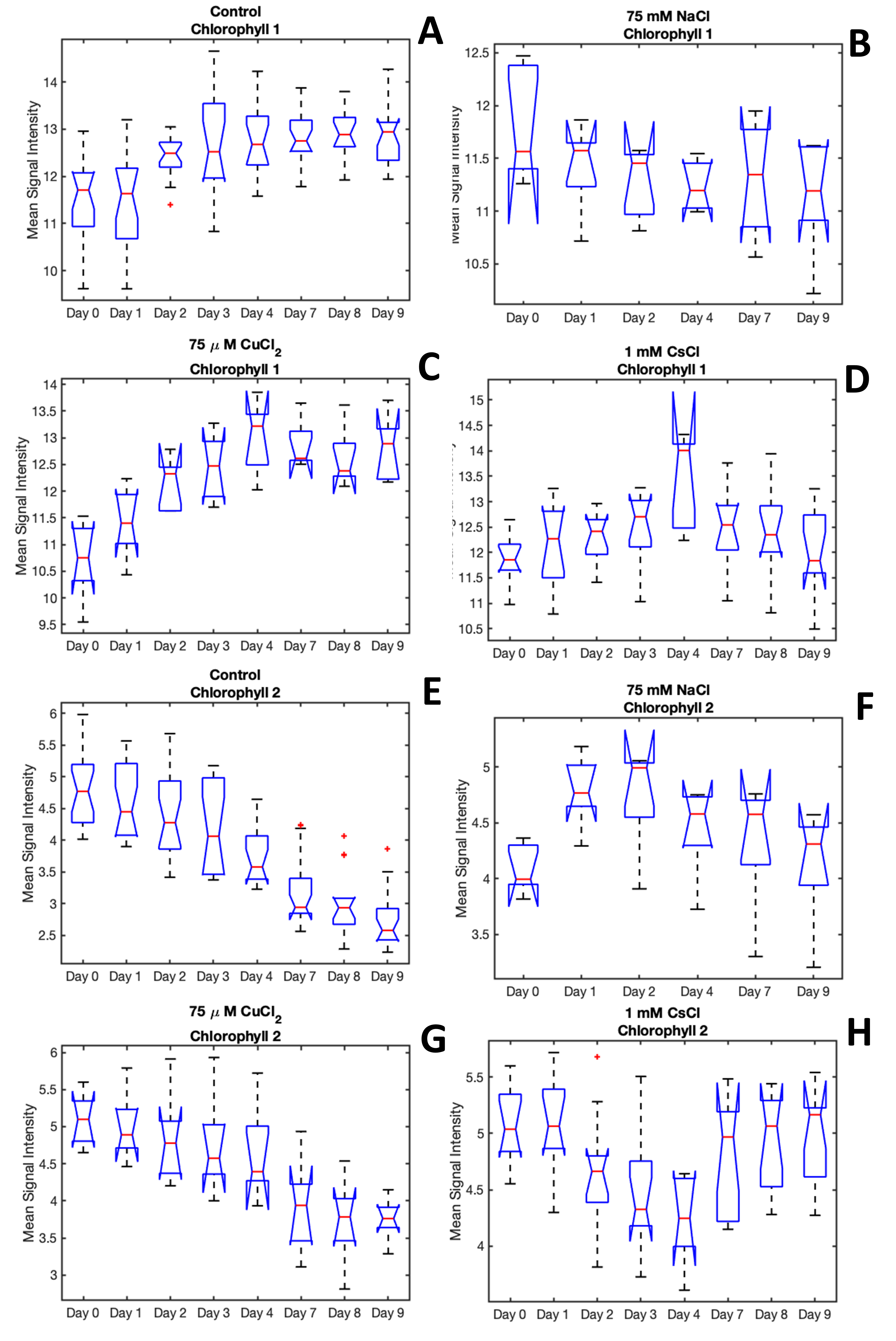

Supplement: Supplementary file 5 [file Image_6.tif]

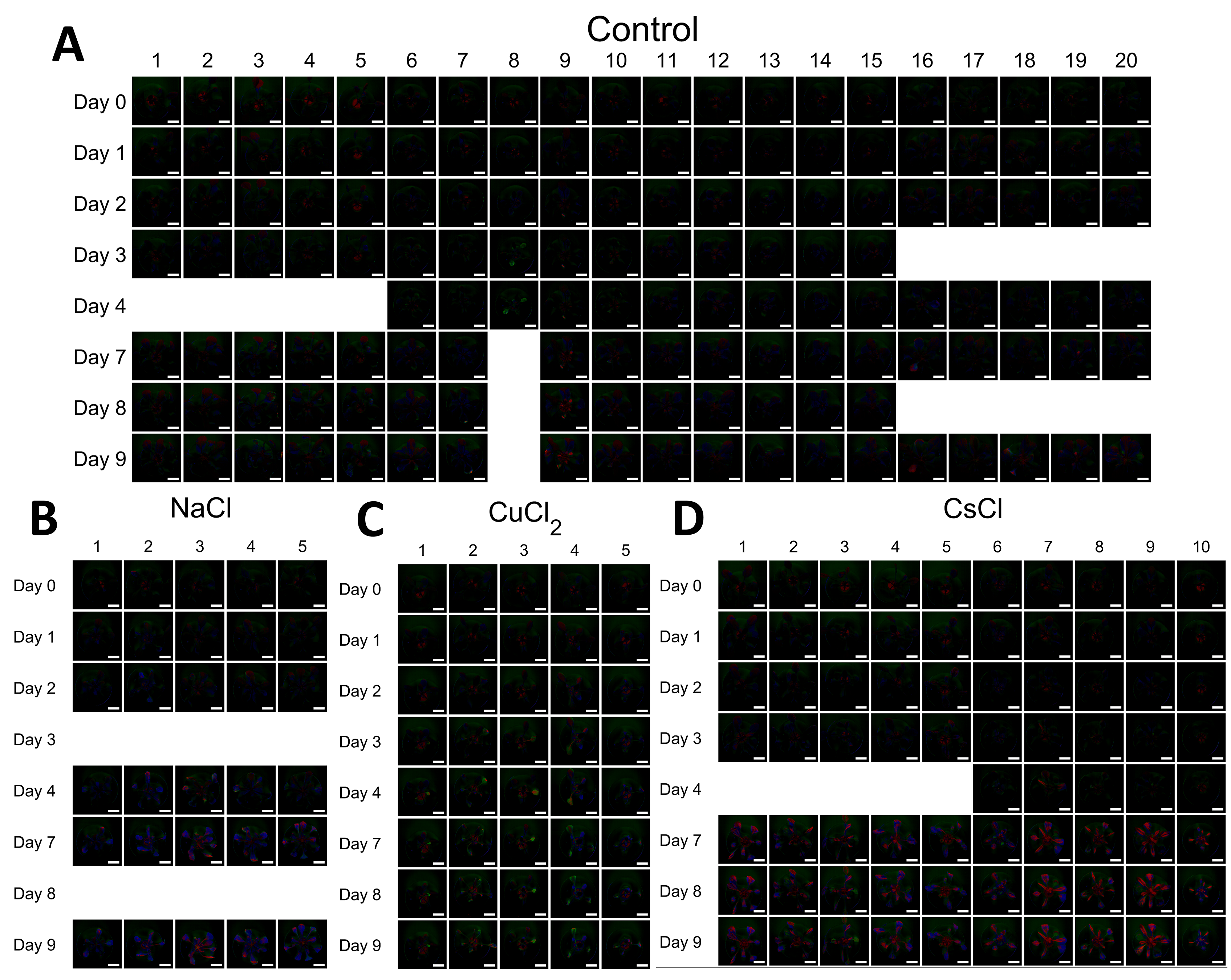

Supplement: Supplementary file 6 [file Image_7.tif]

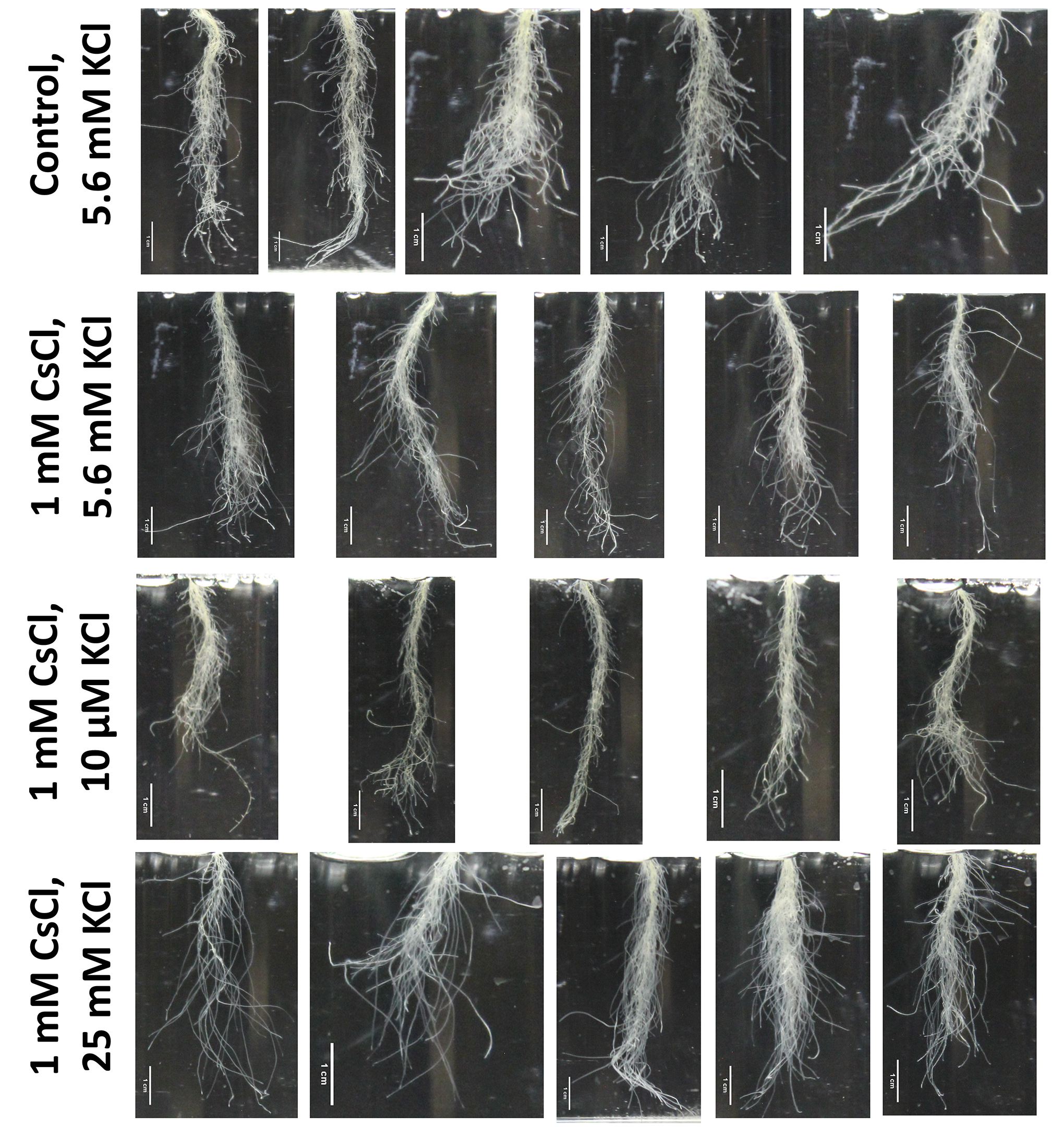

Supplement: Supplementary file 10 [file Image_11.tif]

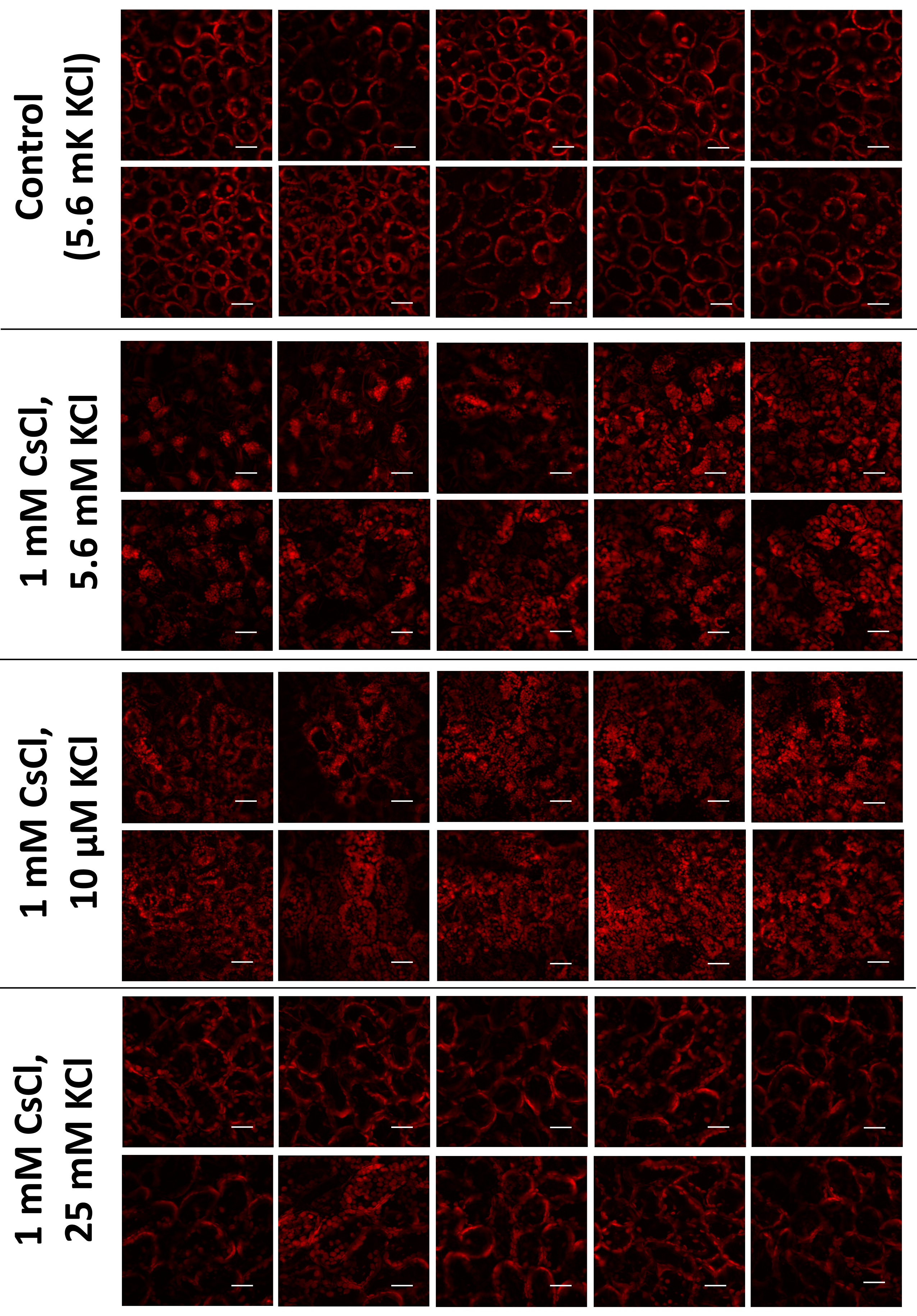

Supplement: Supplementary file 11 [file Image_12.tif]
